# Role of the Lipoperoxidation Product 4-Hydroxynonenal in the Pathogenesis of Severe Malaria Anemia and Malaria Immunodepression

**DOI:** 10.1155/2015/638416

**Published:** 2015-04-19

**Authors:** Evelin Schwarzer, Paolo Arese, Oleksii A. Skorokhod

**Affiliations:** Department of Oncology, University of Torino, Via Santena 5 Bis, 10126 Torino, Italy

## Abstract

Oxidative stress plays an important role in the pathogenesis of *falciparum* malaria, a disease still claiming close to 1 million deaths and 200 million new cases per year. Most frequent complications are severe anemia, cerebral malaria, and immunodepression, the latter being constantly present in all forms of malaria. Complications are associated with oxidative stress and lipoperoxidation. Its final product 4-hydroxynonenal (4-HNE), a stable yet very reactive and diffusible molecule, forms covalent conjugates with proteins, DNA, and phospholipids and modulates important cell functions at very low concentrations. Since oxidative stress plays important roles in the pathogenesis of severe malaria, it appears important to explore the role of 4-HNE in two important malaria complications such as malaria anemia and malaria immunodepression where oxidative stress is considered to be involved. In this review we will summarize data about 4-HNE chemistry, its biologically relevant chemical properties, and its role as regulator of physiologic processes and as pathogenic factor. We will review studies documenting the role of 4-HNE in severe malaria with emphasis on malaria anemia and immunodepression. Data from other diseases qualify 4-HNE both as oxidative stress marker and as pathomechanistically important molecule. Further studies are needed to establish 4-HNE as accepted pathogenic factor in severe malaria.

## 1. Introduction 

In malaria pathophysiology, oxidative stress plays an important role in many fatal endpoints of the disease [1, 2].

Imbalance in redox metabolism may be important under two aspects. On the one hand, prooxidative reactions are important in the host response to combat malaria infection in a controlled manner. Some protective mechanisms against malaria, such as glucose-6-phosphate dehydrogenase- (G6PD-) deficiency [3, 4] and hemoglobinopathies, were proposed to be connected with oxidative stress (reviewed in [1, 2]). Reactive nitrogen species were claimed to have a protective role against blood-stage malaria [5]. However, such protective role has been denied in other studies [6]. On the other hand, excess oxidative stress is harmful for the host and may contribute to malaria complications with potentially fatal outcome, such as severe malaria anemia and immunodepression. Indications for excess lipoperoxidation have been shown in malaria, where plasma lipid peroxides are increased [7] and red blood cells (RBCs) displayed increased lipid peroxidation and decreased antioxidative defense [8, 9] in clinical malaria.

Cellular dysfunctions following excess oxidative stress are frequently mediated by lipoperoxidation products of nonenzymatic degradation of polyunsaturated fatty acids (PUFAs). Lipid peroxidation progresses by free radical chain reactions with lipid hydroperoxides as immediate unstable products that decompose to a series of very reactive products. Among these products, hydroxyaldehydes like 4-hydroxynonenal (4-HNE) are particularly important because they reach relatively high concentrations, are more stable as radicals, and are able to diffuse inside or even outside the cell to reach distant targets. Target molecules, with which 4-HNE forms covalent conjugates, are proteins, DNA, and phospholipids [10, 11].

Thus, 4-HNE can be considered the final mediator and marker of oxidative stress in cells and whole organisms. The role of 4-HNE in malaria pathogenesis was recently considered, and the malaria anemia is under special focus, since 4-HNE was shown to play a pathomechanistic role in this potentially deadly complication.

## 2. 4-HNE Chemistry (Generation, Biologically Relevant Properties, and Metabolism)

**Figure 1:**
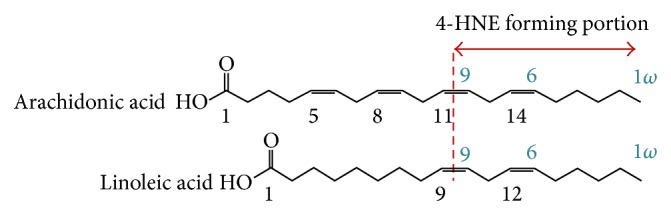
Omega-6 PUFAs are the source of 4-hydroxynonenal (4-HNE).

4-HNE is the final product of two sequential processes: first the generation of the hydroperoxide omega-6 polyunsaturated fatty acid (PUFA) during lipid peroxidation and second the carbon-chain break of the peroxidized PUFA together with the introduction of a hydroxyl group. Only omega-6 PUFAs, such as the essential fatty acids linoleic acid and arachidonic acid, play a role in 4-HNE generation (Figure 1).

Lipid peroxidation is a powerful nonenzymatic chain reaction that continuously provides free lipid radicals for further peroxidation. PUFAs are attacked by free radicals regardless of whether the fatty acid is in free form (e.g., in the circulation, noncovalently bound to albumin) or esterified in phospholipids in the cellular membranes [12].

**Figure 2:**
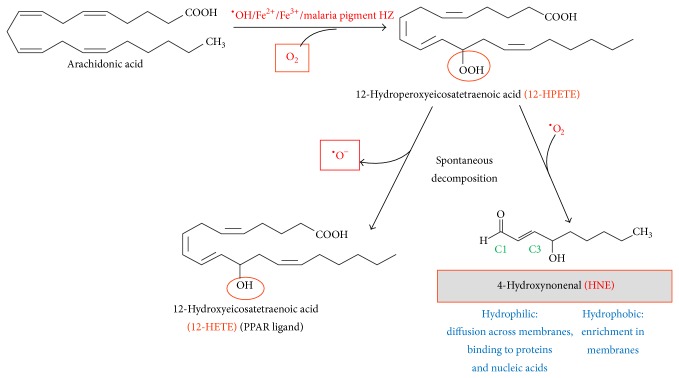
The formation of 4-HNE from arachidonic acid by lipid peroxidation, chain break, and hydroxylation. In malaria, it was shown that the heme core of hemozoin (HZ, malaria pigment) could catalyze the lipoperoxidation of PUFAs. Resulting hydroperoxides are chemically unstable: they (i) are reduced to the hydroxy-fatty acid such as hydroxyeicosatetraenoic acid (HETE) from arachidonic (note, HETEs are bioactive PPAR-*γ* (peroxisome proliferator-activated receptor gamma) ligands) or (ii) are decomposed to 4-HNE. C1 and C3 are 4-HNE reactive sites. Biologically relevant properties of 4-HNE are listed in blue.

Thanks to their unpaired electron, free radicals are highly reactive compounds. One of the most reactive representatives of oxygen radicals is the hydroxyl radical (^•^OH) that is able to initiate the peroxidation process by hydrogen abstraction from a C-atom positioned between two conjugated double bonds in PUFAs (Figure 2). Similarly, iron (Fe^2+^/Fe^3+^) can act as a catalyst for this step and produce additional hydroxyl radicals. The result of hydrogen abstraction is a PUFA radical which binds molecular oxygen, becoming highly reactive peroxide that propagates the peroxidation chain reaction by “stealing” hydrogen from a further PUFA to become a PUFA hydroperoxide (PUFA-OOH). The newly formed PUFA radical may continue the peroxidation chain reaction. Similar to free iron, iron-containing heme is a highly efficient catalyst and accelerates substantially the lipoperoxidation process. The peroxidized PUFA rearranges the hydroperoxy group within the molecule (Hock rearrangement [13]), and the bond between carbons 9 and 10 of the fatty acid chain is destabilized and breaks up. The so-called Hock cleavage splits the peroxidized PUFA molecule between the C11 and C12 carbon atoms (in case of arachidonic acid) and the rearranged peroxy-group and releases the aldehyde nonenal and the oxidized residual 11-C fatty acid. Nonenal consists of the last 9 carbons of the omega-6 fatty acid. A further peroxidation with hydrogen abstraction and oxygen binding occurs at position 4 and the resulting 4-hydroperoxynonenal is finally reduced to 4-HNE. All reactions may run efficiently without enzyme catalysis and are enhanced by heme.

4-HNE is a 9 C-atom long aldehyde with a double bond between the carbon atoms C2 and C3 conjugated to the head aldehyde group (C1) and a hydroxyl group at the C4 position (Figure 2). While the aldehyde group is hydrophilic, the nonpolar carbon chain from C5 to C9 is hydrophobic. This explains the preferential enrichment of the molecule in lipid-rich structures like membranes as well as the capacity to cross the cell membrane. Thus, 4-HNE produced in a cell is enriched intracellularly but is not confined to the cell of origin but is found also extracellularly. 4-HNE was described to interfere with membrane fluidity and phospholipid asymmetry [14, 15]. The molecule is highly reactive due to two reactive sites, the C3 carbon atom and the aldehyde group. First, the conjugated system of C=C double bond and C=O carbonyl group plus the hydroxyl group makes the C3 atom more electropositive and thus susceptible to nucleophilic attacks, which allow the formation of Michael adducts with cysteine, histidine, and lysine residues, and less frequently with arginine residues of polypeptide chains. Second, the aldehyde group may react with free amino group of lysine residues forming so-called Schiff bases frequently seen in lipoproteins. Due to the presence of the two reactive sites in 4-HNE mentioned before, high concentrations of 4-HNE can also cross-link two peptide chains (reviewed in [16]). Further, 4-HNE was described to form adducts with purine bases in DNA (4-HNE-deoxyguanosine). This modification may play a role in tumor pathogenesis [17, 18]. The second nonprotein targets of 4-HNE-conjugation are phosphatidylethanolamine and phosphatidylserine that react with their free amino groups forming Michael adducts and Schiff bases [19–21]. Also vitamin C (ascorbic acid) and pyridoxamine, the precursor of vitamin B6, are targets for 4-HNE-conjugation. Ascorbylation is assumed to play a role in the elimination of 4-HNE from the body [22, 23].

In parallel, unconjugated 4-HNE is metabolized for detoxification and better excretion. The main enzymes involved in 4-HNE metabolism are glutathione-S-transferase (GSTA4-4, EC 2.5.1.18) that catalyzes the synthesis of the 4-HNE-GSH adducts, aldehyde reductase (EC 1.1.1.21) that oxidizes 4-HNE to hydroxynonenoic acid (HNA), and alcohol dehydrogenase (EC 1.1.1.1) that reduces 4-HNE to 1,4-dihydroxynonene (DHN) [24, 25].

Proteins modified by 4-HNE are degraded by the proteasome in the cell cytosol but accumulate in case of high oxidative stress and may contribute to functional losses [26].

## 3. 4-HNE Role in Health and Disease

### 3.1. Cellular Targets of 4-HNE and Its Potential Functional Importance

The concentrations of 4-HNE detectable in human serum under nonpathological conditions are 0.05–0.15 micromoles per liter, increasing with age. Kidney tubular cells, hepatocytes, and monocytes revealed higher tissue concentrations (between 1 and 100 micromoles per liter) varying by tissue type and organism condition (for review see [10, 27]). Although it seems unlikely that overall concentration may reach 100 micromoles per liter in organs, it is plausible that such levels may build up locally near peroxidized membranes. 4-HNE was calculated to reach 4 millimoles per liter in microsomes. Due to cumulative effect in the membrane, 4-HNE may attack critical target proteins in or near the lipid bilayer. Accordingly, monocytes where the enzyme NADPH-oxidase produces oxygen radicals directed against extracellular and intracellular microbes during the oxidative burst reaction may contain 40 micromoles per liter or higher 4-HNE concentrations [28, 29]. At low concentration, 4-HNE is considered an efficient signaling molecule, whilst under oxidative stress the metabolism of 4-HNE may be saturated and modifications of biomolecules may elicit pathological effects. Protein modifications represent the main known mechanism by which 4-HNE influences physiological and pathological processes.

A high and increasing number of 4-HNE target proteins have been identified. The binding sites of 4-HNE to the protein chain were determined by mass spectrometry for about half of the proteins functionally modified by 4-HNE. The functional annotation of the structurally and/or functionally modified proteins covers a wide spectrum including enzymes in energy metabolism, protein kinases and phospholipases involved in cellular signaling, enzymes involved in intracellular proteolysis by the proteasome, extracellular matrix metalloproteinases, membrane receptors for neurotransmitter and growth factors, membrane ion transporter and carriers in the plasma membrane, some plasma proteins, cytoskeletal proteins, heat-shock proteins, and proteins involved in translation [10, 27, 30–35].

### 3.2. Regulation of Physiological Processes

The cellular increase of 4-HNE in response to oxidative stress can potently activate stress response mechanisms. Nontoxic levels of 4-HNE have been described to modify (i) signal transduction regulating the* de novo* synthesis of detoxifying enzymes (e.g., via transcription factor Nrf2 and the transcription-enhancer antioxidant response element ARE); (ii) cell proliferation (e.g., via c-Jun N-terminal kinase JNK); (iii) apoptosis and cell cycle regulation (e.g., via p53); (iv) differentiation (e.g., via mitogen-activated protein kinase MAPK and growth factor receptors); (v) inflammatory response; (vi) expression of the cellular adhesion molecules ICAM-1, VCAM-1, and E-selectin (e.g., via transcription factors p38 and NF-kappaB); and (vii) protein turnover (via proteasome activity) [10, 11, 36–38]. Modification of these pathways may be associated with survival mechanisms of cells or tissues under stress condition.

### 3.3. Association of 4-HNE with Pathologies

Uncontrolled or excessive production of 4-HNE could interfere with basal cellular signaling and play a role in the pathogenesis of several diseases accompanied by oxidative stress. In 39 different human diseases increased steady-state levels of 4-HNE have been either reported in the affected organ or generalized in the blood plasma or both [10, 39] which suggests a pathogenic role of the aldehyde in the disease pattern and progression. It is now accepted that 4-HNE plays a role in inflammation-related cell signaling. Importantly, inflammation represents the main driving force in progression of most chronic diseases, in acute inflammatory diseases and in some anemias, as renal anemia [40].

## 4. 4-HNE Production in Malaria

4-HNE has received little attention by malariologists as yet, although elevated levels of free, unconjugated 4-HNE had been described in RBCs of* P. vinckei*-infected mice as early as 1988 [41]; one study reported the growth inhibition of* in vitro* cultured parasites by 4-HNE [42], and the general presence of oxidative stress in malaria was detected long ago [43–46].

### 4.1. 4-HNE from the Parasite

**Figure 3:**
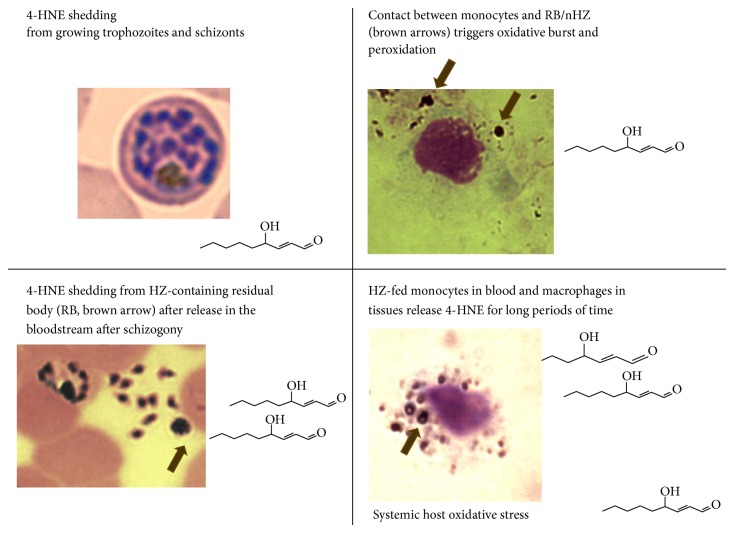
4-HNE source in malaria.

**Figure 4:**
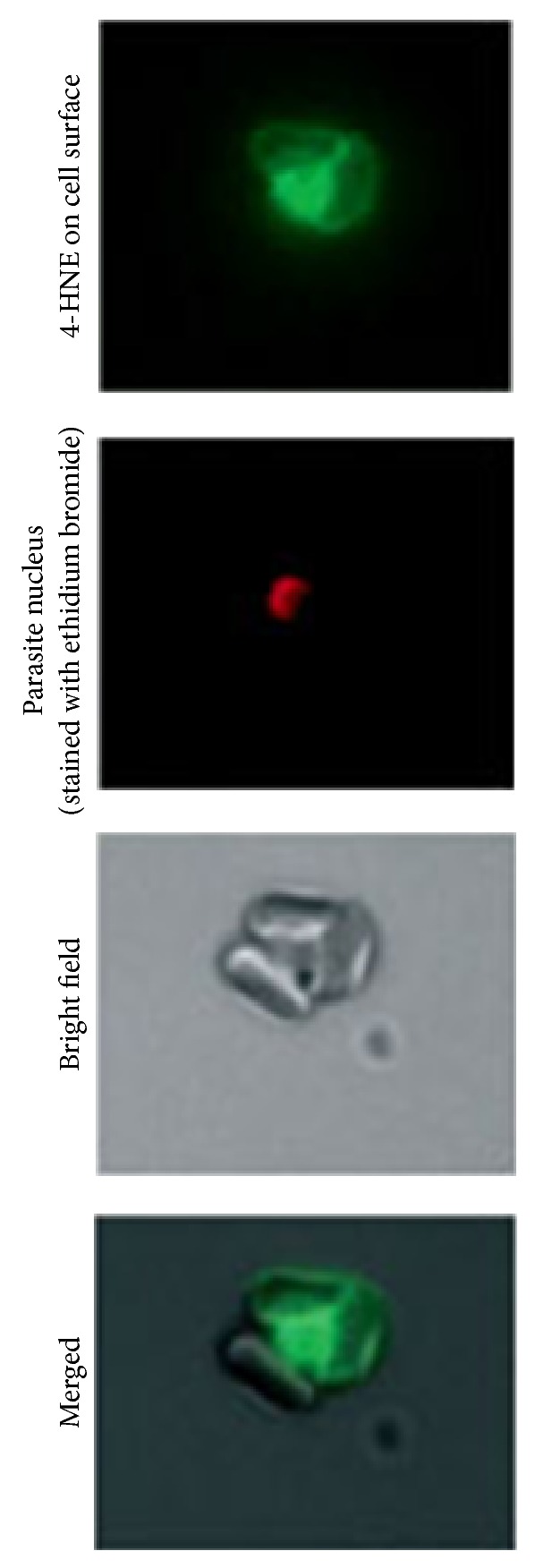
4-HNE transfer from parasitized to nonparasitized RBCs in rosettes* in vitro*. Micrographs of 4-HNE-positive parasitized RBCs and nonparasitized RBCs. This result was originally published in British Journal of Haematology, Blackwell Publishing Ltd. (Uyoga et al. 2012, © John Wiley & Sons Ltd.).

During its development the malaria parasite digests the major part of host RBC hemoglobin and polymerizes the undigested and prooxidant heme to HZ (hemozoin, malaria pigment) in close contact with the membrane of the digestive vacuole of the parasite [47]. Similar to free heme, isolated HZ catalyzes* in vitro* the peroxidation of PUFAs with subsequent degradation to 4-HNE and other products (Figures 1 and 2 and [48]). The onset of hemoglobin degradation and HZ formation at young trophozoite stage coincided with a significantly enhanced formation of 4-HNE and 4-HNE-membrane protein conjugates in parasitized RBCs, compared to nonparasitized control RBCs. The progressive HZ generation during parasite growth was accompanied by the increase of 4-HNE conjugates up to 15-fold higher levels in HZ-rich schizonts compared to young HZ-free ring-forms (Figure 3). The conjugates can be detected and quantified by flow cytometry or fluorescence microscopy after recognition by specific antibodies [11, 49, 50]. 4-HNE conjugates are not confined to 4-HNE-producing cells but were also found in nonparasitized RBCs (npRBCs) cytoadherent to trophozoites (see Figure 4) [51].

### 4.2. 4-HNE from the Residual Body

**Figure 5:**
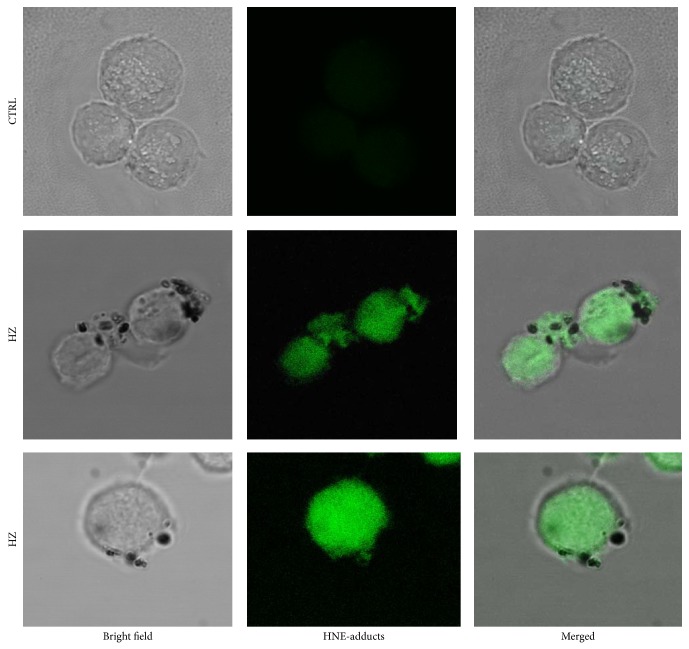
4-HNE conjugates on the cell surface of erythroid cells cocultivated with residual body (RB or natural HZ) visible as green fluorescence. This research was originally published in Blood (Skorokhod et al. 2010, supplemental materials© the American Society of Hematology).

The synthetic beta-hematin core of natural HZ (nHZ) is able to catalyze* in vitro* the PUFA peroxidation with formation of 4-HNE and hydroxy-fatty acids such as hydroxylated arachidonic acid (hydroxyeicosatetraenoic acid, HETE) or hydroxylated linoleic acid (hydroxyoctadecadienoic acid, HODE) (Figure 2, [48]). At the end of the 48 h parasite growth cycle in the RBC, the daughter parasites leave the RBC to infect fresh RBCs (schizogony). In parallel the residual body (RB) or nHZ, that is, the membrane-enveloped, HZ-containing digestive vacuole, is expelled into the bloodstream. The RBs collected from culture supernatant contained 50 micromoles per liter of free 4-HNE (Figure 3, [52]), which was released and transferred to adjacent cells in an* in vitro* erythropoiesis model (Figure 5). Hence, RBs represent an additional source of 4-HNE in pathology [52].

### 4.3. 4-HNE from Human Phagocytes

RBs released into plasma bind preferentially host fibrinogen and to a lesser extent immunoglobulins (IgGs) and complement factors. Fibrinogen was constantly present at 10–100,000 fibrinogen molecules per residual body and was stably bound to nHZ collected from plasma-cultured parasites [53]. Surface-binding changes the conformation of fibrinogen and makes it recognizable by Toll-like receptor 4 (TLR4) on monocytes. RBs coated with fibrinogen become excellent ligands for TLR4 and were responsible for the rapid 100-fold stronger production of reactive oxygen species by human monocytes [53]. Close contact of HZ with the cell membrane, together with extensive oxidative burst during HZ phagocytosis, resulted in fast and persistent 6-fold increase of lipid peroxides in RB-fed monocytes compared to the transient 2-fold increase after RBC phagocytosis [54]. 4-HNE was significantly increased at 2 h after feeding monocytes with HZ and peaked at 5 h attaining 50-fold levels compared to unfed controls and was still almost 15-fold the control levels after 12 h [28]. The long-lasting generation of 4-HNE (Figure 3) is caused by a persisting lipoperoxidation catalyzed by HZ after its uptake into the lysosome of the monocytes. The generation of 4-HNE in HZ-fed monocytes is followed by a significant increase of 4-HNE conjugate formation with membrane proteins [52].

In summary, HZ can be considered to be source and shuttle for 4-HNE in malaria and 4-HNE is likely one molecular mediator of the HZ effects (reviewed in [55, 56]).

## 5. 4-HNE Effect on Immune Response and Malaria Anemia

HZ or RB has been described to interfere with several immune processes that might play a role in malaria and contribute to the lack of sterile immunity as well as in complicated forms of malaria [56]. As described above, HZ acts as catalyst and shuttle for 4-HNE in malaria. Thus, a series of HZ effects were recapitulated by exogenously added 4-HNE, and molecular targets for 4-HNE listed above were modified by 4-HNE in HZ-fed cells. The roles of 4-HNE on the pathophysiologically relevant processes are listed below.

### 5.1. Phagocytosis, Oxidative Burst, and MHC Class II Expression: Protein Kinase C (PKC) Involvement

Phagocytosis and oxidative burst are first-line defense mechanisms against the blood-stage malaria parasite. After avid phagocytosis of HZ and a violent oxidative burst elicited by mature parasites and released RB, both phagocytosis and burst are switched off after approximately 6–12 h from initial phagocytosis and remain inactive during the observation period of 3 days [54]. Phagocytes do not die and do not show signs of apoptosis [54, 57] that could explain the inactivation. In malaria disease large proportions of resident macrophages and circulating monocytes and neutrophils contain massive amounts of HZ. When fed with HZ* in vitro*, the precursors of macrophages, the monocytes, lose their capacity to upregulate the antigen presenting MHC class II in response to interferon-gamma suggesting that HZ loading may contribute to the impairment of the immune response and derangement of antigen presentation previously reported in* falciparum* malaria [56, 58].

Protein kinase C (PKC), the common regulator protein that coordinates phagocytosis, burst, and antigen presentation, was shown to be inactivated by 4-HNE-conjugation* in vitro*, and HZ-fed monocytes exhibited a high level of 4-HNE-conjugated PKC [28]. The kinetics of 4-HNE production and conjugate formation after HZ phagocytosis fit with the quick loss of active membrane-associated PKC and the subsequent disappearing responsiveness of cytosolic PKC to activators in HZ-fed monocytes [59]. Thus, we consider 4-HNE to be a molecular mediator in the HZ-provoked modulation of immune responses such as inhibition of oxidative burst, phagocytosis, and antigen presentation by MHC class II.

Comparison of transcriptional changes elicited by synthetic HZ and 4-HNE in murine macrophages suggests that both are involved in the modulation of inflammatory responses, transcription factor NF-kappaB-dependent signal transduction, and extracellular matrix degradation [60] further confirming 4-HNE as an immune-modulating factor of interest in malaria.

### 5.2. Dendritic Cell Differentiation and Maturation

Enhanced 4-HNE production and formation of membrane protein-4-HNE conjugates were observed in HZ-fed monocytes in their differentiation towards DC [52]. Functionally, phagocytosis of HZ-containing parasites or HZ by itself (HZ is here synonymous with RBs), as well as 4-HNE and/or HETE generated and carried by HZ, inhibited the differentiation and maturation of DC [57]. In particular, 4-HNE significantly inhibited the expression of CD83, the functionally important marker for mature DCs, mimicking the effect of HZ or HETE [56, 57]. Moreover, 4-HNE dose dependently inhibited the expression of CD83 in differentiating DCs [52] and 4-HNE added at reasonably low doses significantly inhibited the protein expression of MHC classes I and II, CD1a, and accessory molecules, which can modulate the ability of immune cells to develop or perform their functions [61], such as CD40, CD54, and CD83 [57]. Additionally, 4-HNE modified the responsiveness of GM-CSF receptor, the crucial growth factor for dendritic cell differentiation* in vitro* [52].

Similar to phagocytosis of HZ, the addition of 4-HNE resulted in increased expression of the nuclear receptor PPAR-gamma (peroxisome proliferator-activated receptor gamma) in the immune cells [36, 57]. After activation (e.g., by HETE) PPAR-gamma moves into the nucleus where it regulates immune cell functions [62] and functionally suppresses the transcription factor NF-kappaB [63]. NF-kappaB controls proinflammatory immune responses [64], DC differentiation, DC maturation, and CD83 expression related to the latter process.

### 5.3. Cell Cycle Control and Membrane Receptor Expression in Erythropoiesis

Dyserythropoiesis (defective production of RBC) is an important determinant of severe malaria anemia. Erythropoiesis prominently occurs in the bone marrow erythroblastic islands, microenvironmental niches in which erythroid progenitors proliferate, differentiate, and finally expel their nucleus at the end of the maturation process. An island is formed by a central macrophage surrounded by developing and differentiating erythroid cells from early stages through reticulocytes [65]. Marrow macrophages and erythroid precursors have been shown to contain HZ in malaria patients [66, 67]. 4-HNE, the production of which is enhanced by HZ, was first shown to interfere with erythropoiesis in an* in vitro* model of the initial stages of erythroid development in the bone marrow [68]. Both supplemented free 4-HNE and 4-HNE contained in HZ supernatants inhibited erythroid cell colony growth. Application of advanced cell culture protocols allows the complete differentiation of umbilical cord blood-derived erythroid stem cells to fully mature enucleated RBCs and to analyze biochemical parameters crucial for this proliferation-dependent differentiation program. Erythroid stem cells incubated with 4-HNE displayed sustained high levels of 4-HNE-protein conjugate on their surface [69]. Human phagocytes either HZ-laden or cocultured with HZ were able to shed 4-HNE that diffused to the surrounding erythroid cells and was detected on the surface of those cells as 4-HNE-protein conjugate (Figure 5). 4-HNE inhibits the erythroid cell proliferation during the whole period of erythropoiesis, involving the molecular regulators of the cell cycle, a series of controlled events that take place in a cell leading to its division [69]. Both natural HZ and 4-HNE treatment of erythroid cells results in (i) increased regulatory proteins: p53 (tumor suppressor protein 53) and p21 (cyclin-dependent kinase inhibitor 1); (ii) unbalanced phosphorylation of the retinoblastoma protein Rb; (iii) decreased levels of cyclins A and D. Importantly, neither HZ nor 4-HNE added at reasonable concentrations induced programmed cell death (apoptosis). Further, low micromolar 4-HNE delays the differentiation marker expression: the functionally important membrane receptors for transferrin (TfR1, CD71), stem cell factor c-kit (CD117), interleukin-3 (CD123), and erythropoietin (EpoR) as well as the stem cell marker CD34, and the RBC markers glycophorin A and hemoglobin. GATA-1, the master transcription factor that controls the differentiation processes in erythropoiesis, was less expressed in HZ- and 4-HNE-treated cells. The impaired cell cycle and reduced receptor expression decreased the total cell yield and specifically the yield of differentiated erythroid cells [69]. These results reconfirm the inhibitory role of HZ, identify 4-HNE as HZ-generated inhibitory molecule for proliferation and differentiation, and describe molecular targets of 4-HNE in erythroid progenitors possibly involved in the inhibition of erythropoiesis in malaria anemia.

### 5.4. RBC Membrane Proteins and Phagocytosis

Excessive loss of npRBC, in addition to impaired erythropoiesis, is considered to be cause for malaria anemia. Parasitized RBCs generate 4-HNE that conjugates with membrane skeleton proteins such as spectrin and actin and the integral membrane protein band 3 [51, 70] that contributes to anchoring the cytoskeleton to the membrane. 4-HNE-conjugation is not confined to the parasitized RBCs but 4-HNE is transferred and binds to adjacent npRBCs as shown in Figure 4 where the central trophozoite-infected RBC is surrounded by npRBCs [51]. The differential labeling of pRBCs and npRBCs with a nuclear fluorescent probe (e.g., ethidium bromide) allows the separate analysis by flow cytometry of membrane protein-4-HNE conjugates in both cell cohorts [51]. Similar to npRBCs, protein-4-HNE conjugates are detectable in 4-HNE-treated RBCs causing loss in deformability and increased phagocytosis [51]. As decreased deformability and increased phagocytosis add to RBC elimination in the spleen, 4-HNE is suggested to be instrumental for the removal of large numbers of npRBCs.

### 5.5. Cytoskeleton Modifications and Impairment of Chemotactic Motility and Transendothelial Migration of Monocytes

Immune cell motility is an important physiological process, relatively poorly studied in malaria. Recently it was shown that nHZ phagocytosis by human primary monocytes impaired their chemotactic motility toward MCP-1, TNF, and FMLP and their diapedesis across a confluent endothelial cell layer toward MCP-1 [71]. No inhibition was detected in control latex-fed or unfed monocytes. Microscopic analysis has shown that the polarization defect in nHZ-fed monocytes was due to irregular actin polymerization. Similar to nHZ phagocytosis, the exposure of monocytes to* in vivo*-compatible 4-HNE concentrations inhibited cell motility in both the presence and the absence of chemotactic stimuli, suggesting the impairment of cytoskeleton dynamics. Consequently, 4-HNE conjugates with the cytoskeleton proteins *β*-actin and coronin-1A were identified in nHZ-fed monocytes and localized in domains of protein-protein interactions involved in cytoskeleton reorganization and cell motility. The molecular and functional modifications of actin and coronin by nHZ/4-HNE may also explain impaired phagocytosis, another motility-dependent process previously described in nHZ-fed monocytes [54]. This study indicates that impaired monocyte motility may contribute to the immune depression and the frequent occurrence of secondary infections observed in malaria patients [71].

**Figure 6:**
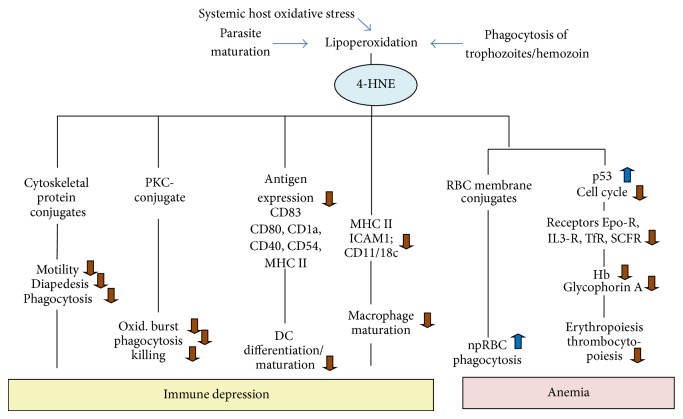
Proteins and processes of potential relevance for malaria pathology structurally or functionally modified by 4-HNE.

Figure 6 summarizes molecules and processes of potential relevance for malaria pathology that were structurally or functionally modified by 4-HNE.

## 6. 4-HNE in Clinical Malaria and in Malaria Anemia

*In vitro* and clinical data strongly indicate that oxidative stress plays important roles in* falciparum* malaria, notably in the pathogenesis of severe anemia. However, oxidative modifications of the RBC membrane by 4-HNE, which may mechanistically clarify the pathogenesis of clinical malaria as described above, have not been tested for their clinical significance until recent times.

Two important clinical trials, performed from 2005 to 2011 in malaria endemic regions of Mozambique [9] and Kenya [51], have shown that increased 4-HNE generation is occurring in clinical malaria. Small cohorts with mild to severe anemia have been studied to see whether a correlation between 4-HNE-conjugation and anemia was present.

### 6.1. Elevated 4-HNE Conjugates in Clinical Malaria

Oxidative stress markers including 4-HNE conjugates in RBC membranes and advanced oxidation protein products (AOPP) were longitudinally assessed in a large cohort of nonimmune Mozambican newborns. The study was performed in the period from 2.5 to 24 months of age, and additionally at the first clinical malaria episode and in convalescence, and included analysis of association with the malaria incidence rate until 2 years of age [9]. A large data base for control values and values in clinical malaria of the same cohort of very young children allows defining the influence of clinical disease on 4-HNE generation: (i) 4-HNE conjugates significantly increased by approximately 75% in parasitized and nonparasitized RBCs during clinical malaria; (ii) 4-HNE conjugates correlated with plasma but not with RBC oxidative stress markers during clinical malaria; (iii) in contrast to plasma oxidative stress markers, 4-HNE conjugates remained significantly elevated in convalescence after clearing of malaria parasites from peripheral blood in comparison to the healthy state before clinical malaria; and (iv) high levels of 4-HNE conjugates in individual healthy children were predictive of increased malaria incidence rates until 2 years of age [9]. Above data underscore the clinical significance of 4-HNE in malaria immunology and the potential role of 4-HNE for impaired immune functions of phagocytes as described in Sections 5.1 and 5.2.

### 6.2. Role of 4-HNE in Malaria Anemia

**Figure 7:**
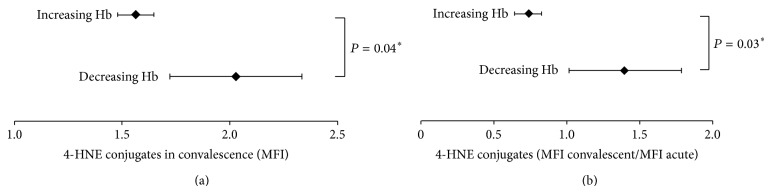
Association of 4-HNE conjugate levels on RBCs with sustained moderate anemia during convalescence. Children presenting with Hb < 100 g/L at their first acute malaria episode were grouped according to their capacity to recover from anemia by increasing Hb levels during convalescence or to worsen their state by decreasing or sustained low Hb levels during convalescence (1 month after acute malaria episode). 4-HNE binding to the RBCs of children was assessed as MFI at acute and convalescence states. Mean MFI-values ± SE during convalescence are plotted for the groups with increasing Hb (+35, 3 ± 6,5%; *N* = 34) and decreasing Hb (−15, 4 ± 6,0%; *N* = 8) (a). The change in 4-HNE conjugate levels in the RBC membrane during recovery from malaria disease is shown (b). The mean ± SE of the ratio of 4-HNE conjugates measured during convalescence and at acute state is plotted for the increasing (+38,5 ± 9,2%; *N* = 22) and decreasing (−26,8 ± 6, 2%; *N* = 4) Hb groups, respectively (b). The significance of differences between groups is indicated by ∗ when *P* < 0.05. MFI, mean fluorescence intensity; Hb, hemoglobin. This result was originally published in British Journal of Haematology, Blackwell Publishing Ltd. (Aguilar et al. 2014, © John Wiley & Sons Ltd.).

As outlined before, 4-HNE demonstrated a strong inhibitory effect on* ex vivo* erythropoiesis of human erythroid stem cells, also contributing to enhanced phagocytosis of RBCs* in vitro*. Both erythropoiesis inhibition and enhanced phagocytosis of npRBCs were shown to contribute to severe malaria anemia. First indications for the clinical relevance of 4-HNE as a molecular pathogenic factor for malaria anemia come from studies in Mozambique and Kenya mentioned before [9, 51]. Elevated or increasing 4-HNE conjugate levels in convalescence were associated with sustained anemia or aggravated anemia during convalescence (Figure 7, [5]). Children with severe malaria anemia had significantly higher percentages of 4-HNE-conjugate-positive npRBCs compared to children with uncomplicated malaria [51]. In conclusion, 4-HNE is suggested to play a role in the phagocytic removal of npRBCs, one hallmark of severe malaria anemia.

Of note, field studies have shown that another blood oxidative stress marker, the advanced oxidative protein products (AOPP), has been shown to be predictive of malaria anemia [72]. This data, together with the correlation between AOPP and 4-HNE conjugates level on RBC established in clinical malaria [9], will strengthen the role of increased 4-HNE levels as part of systemic oxidative stress.

## 7. Conclusions

Results of studies summarized in this review indicate that 4-HNE is a highly active molecule produced by the malaria parasite and by the host immune system in response to infection. In* ex vivo* cell cultures 4-HNE was found to impair several immune functions of phagocytic cells that certainly contribute to malaria pathology. Similarly, 4-HNE was found to impair the development of mature RBCs and to enhance their elimination by the spleen leading to anemia. Clinically, malaria disease is accompanied by elevated 4-HNE levels. Clinical studies indicate that 4-HNE is involved in the pathogenesis of malaria anemia. However, despite abundant evidence from current research, further clinical studies are necessary to better establish this promising molecule as an accepted pathogenic factor in severe malaria.
